# Leg length discrepancy before total knee arthroplasty is associated with increased complications and earlier time to revision

**DOI:** 10.1186/s42836-023-00221-3

**Published:** 2024-01-16

**Authors:** Kevin L. Mekkawy, Ty Davis, Philip A. Sakalian, Alejandro E. Pino, Arturo Corces, Martin W. Roche

**Affiliations:** 1 Hospital for Special Surgery, West Palm Beach, FL 33401 USA; 2 South Shore University Hospital, Bay Shore, NY 11706 USA; 3 Holy Cross Orthopedic Institute, Holy Cross Health, Fort Lauderdale, FL 33334 USA; 4 Department of Orthopaedic Surgery, Larkin Community Hospital, South Miami, FL 33143 USA

**Keywords:** Leg length discrepancy, Total knee arthroplasty, Complications, Outcomes

## Abstract

**Introduction:**

Leg length discrepancy (LLD) following total knee arthroplasty (TKA) is a common complaint, leading to decreased patient satisfaction. However, the effect of LLD diagnosis prior to TKA on outcomes and complications is not well defined. Thus, this study aimed to assess the effects that LLD has on rates of falls and implant complications, length of stay and readmissions, and implant survivorship following TKA.

**Methods:**

A retrospective review of a private insurance claims database was conducted from 2010 to 2021. All cases of TKA and those with a diagnosis of leg length discrepancy were identified. Patients undergoing TKA with a diagnosis of LLD were matched to control patients 1:5 based on demographic and comorbidity profiles. Two-year fall rates and implant complications, lengths of stay, 90-day readmissions, and time to revision were compared between cohorts.

**Results:**

A total of 1,378 LLD patients were matched to 6,889 control patients. The LLD group had significantly higher rates of falls, dislocation, mechanical loosening, periprosthetic fracture, and fibrosis when compared to the control group (all *P* < 0.01). Additionally, mean length of stay was significantly greater in the LLD group (4.9 days vs. 3.0 days, *P* < 0.001). There was no significant difference in 90-day readmission rates between groups (*P* = 0.178). Time to revision was significantly shorter in the LLD group (392 days vs. 928 days, *P* < 0.001).

**Conclusions:**

Leg length discrepancy in patients undergoing TKA was associated with significantly increased fall risk, rates of implant complications, length of stay, and faster time to revision. The findings of this study may allow orthopedic surgeons to identify those patients at risk and allow for more educated patient counseling and operative planning.

**Level of evidence:**

III, retrospective case–control study.

**Graphical Abstract:**

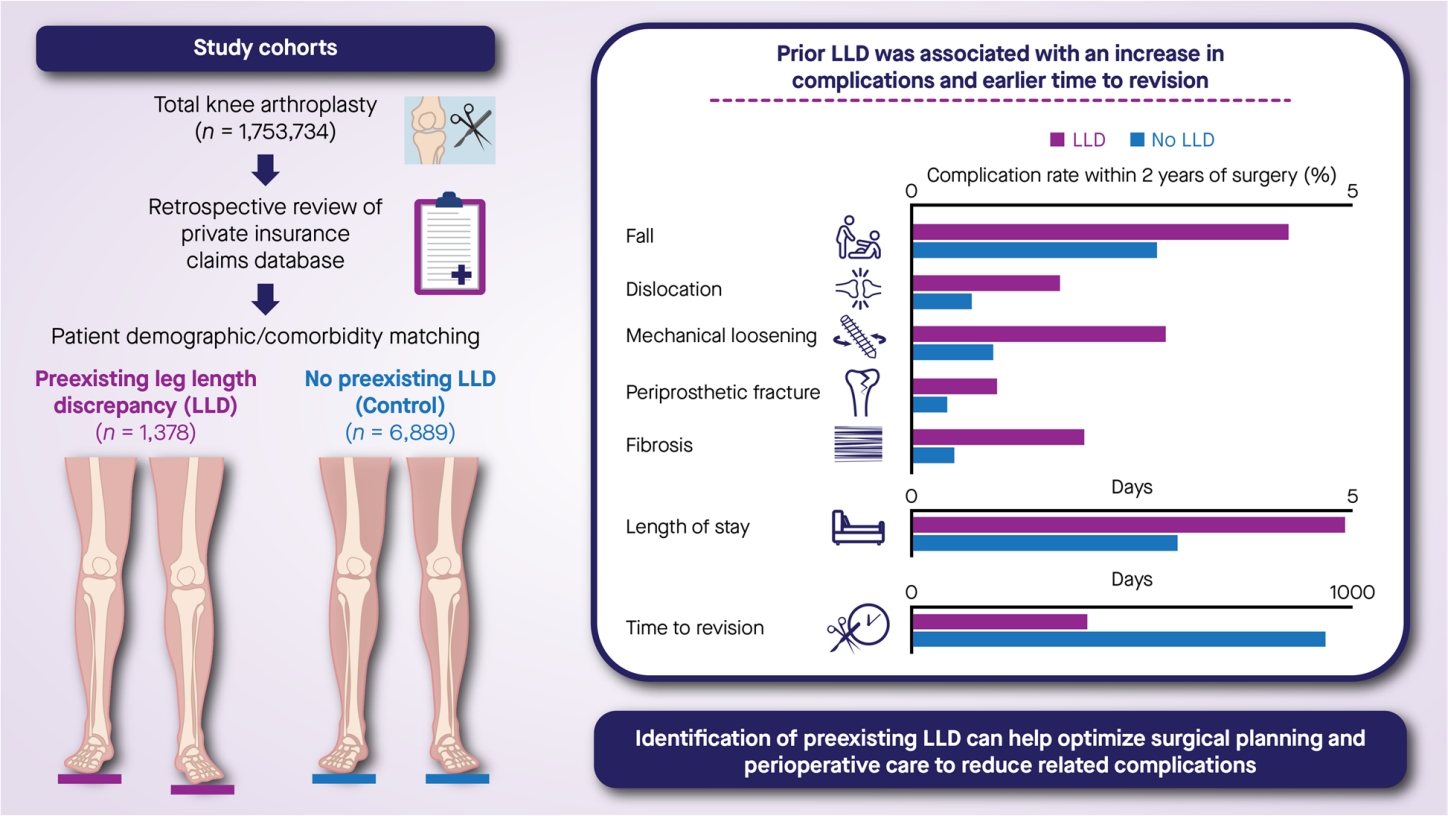

**Supplementary Information:**

The online version contains supplementary material available at 10.1186/s42836-023-00221-3.

## Introduction

Leg length discrepancy (LLD) is a condition where one leg is shorter or longer than the other, possibly resulting in an unequal distribution of weight and altered biomechanics. LLD can occur naturally or be a consequence of various factors such as trauma, infection, or prior surgeries [1]. This condition can increase stress on the joints and accelerate joint degeneration and increase the risk of developing osteoarthritis [2]. Total knee arthroplasty (TKA) is the gold standard for the treatment of end-stage osteoarthritis, with the goal of improving patients’ pain, function, and quality of life. Patients presenting for evaluation for TKA may have an existing LLD due to an altered hip-knee-ankle angle or excessive varus or valgus deformity of the lower extremity [3]. Adequate correction of significant LLD during TKA can be challenging due to the importance of ligamentous balance and bony resection which takes priority over length correction [3, 4]. However, there is still a large portion of patients who are dissatisfied following their TKA regarding pain relief and satisfaction [5, 6]. TKA is expected to continually rise and is estimated to reach 3 million cases annually in the United States by 2030 [7]. Therefore, identifying areas of improvement in outcomes as well as patient satisfaction is paramount.

LLD following total hip arthroplasty is well described in the orthopedic literature, with increased postoperative hip pain, lower functional hip scores, decreased patient satisfaction, and higher rates of litigation [8–12]. Unlike total hip arthroplasty, however, LLD is rarely regarded as a significant issue following TKA. Achieving proper limb alignment and length is crucial for optimal function, stability, and patient satisfaction after TKA as well [13]. Significant LLD can lead to gait abnormalities, increased stress on the joints, and potential complications including pain, discomfort, and an increased risk of implant failure [13, 14]. However, preoperative LLD is not commonly assessed in patients with knee osteoarthritis, and its effects on outcomes following TKA are not well-defined in the literature. LLD, especially long-standing, may lead to significant challenges in soft tissue and gap balancing, which may lead to postoperative complications and worse patient-reported outcomes.

The purpose of this study was to assess the effect that prior limb length discrepancy has on (1) fall risk, (2) 2-year prosthetic complications, (3) length of stay and 90-day readmission rates, and (4) incidence of revision knee arthroplasty and time to revision in patients undergoing total knee arthroplasty.

## Methods

This study was deemed exempt by an Institutional Review Board.

We conducted a retrospective review of the Mariner Database within the PearlDiver Database (PearlDiver Technologies, Colorado Springs, CO, USA) from January 2010 to October 2021. PearlDiver is a commercially available database and has been used extensively in orthopedic literature. The data is Health Insurance Portability and Affordability Act (HIPAA) compliant. International Classification of Disease (ICD), the Ninth and Tenth Revision codes, and Current Procedural Terminology (CPT) codes were utilized to identify patients, diagnoses, procedures, and complications. A summary of the codes used is summarized in Supplemental Table 1.

**Table 1:** Demographic and comorbidity characteristics of TKA patients with LLD and a matched-control cohort

Patient Demographic	LLD *n* = 1,378 *n* (%)	Control *n* = 6,889 *n* (%)	*P-*Value
Age^a^	63.9 ± 10.0	63.9 ± 10.0	0.979
Gender			1.000
Female	825 (67.4)	4,125 (67.4)	
Male	553 (32.6)	2,764 (32.6)	
Comorbidity			
Alcohol use	122 (8.9)	610 (8.9)	1.000
COPD	452 (32.8)	2,260 (32.8)	1.000
Diabetes mellitus	599 (43.5)	2,995 (43.5)	1.000
Hypertension	1,166 (84.6)	5,830 (84.6)	1.000
Obesity	821 (59.6)	4,105 (59.6)	1.000
Tobacco use	639 (46.4)	3,195 (46.4)	1.000

*TKA* total knee arthroplasty, *LLD* leg length discrepancy, *COPD* chronic obstructive pulmonary disease

^a^Given as mean ± standard deviation;

**Fig. 1:**
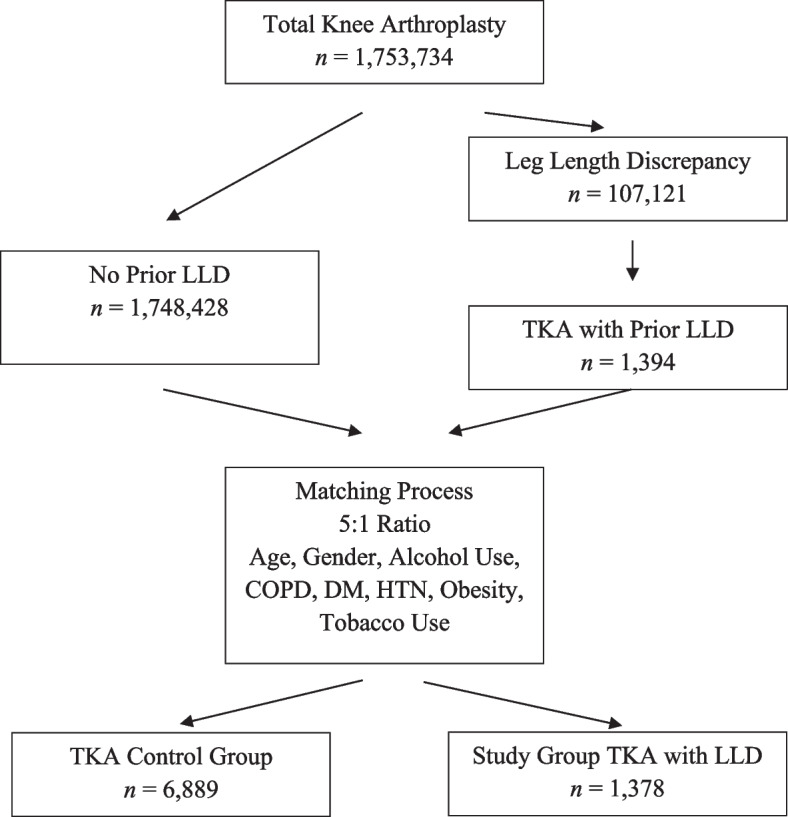
Flow diagram depicting inclusion and matching criteria between TKA patients with prior LLD. LLD, leg length discrepancy; TKA, total knee arthroplasty; COPD, chronic obstructive pulmonary disease; DM, diabetes mellitus; HTN, hypertension

Patients who had a prior diagnosis of leg length discrepancy were first identified using ICD-10 M2175 through M21769. Patients who underwent TKA were identified using CPT-27445 and CPT-27447, ICD-9 81.54, and ICD-10 codes. A cohort was then created including patients who underwent TKA and had a prior diagnosis of leg length discrepancy. This cohort was subsequently matched in a 1:5 fashion to TKA patients with no leg length discrepancy diagnosis, based on demographic and comorbidity profiles. These patient variables for which they were matched included age, gender, alcohol use, tobacco use, diabetes mellitus, chronic obstructive pulmonary disease, obesity, and hypertension. Following the inclusion and matching process, a total of 1,378 patients with a diagnosis of LLD were matched to 6,889 control patients. The results of the matching process were successful, with no significant differences between groups regarding age, gender, and comorbidity burdens (Fig. 1, Table 1).

The primary outcomes included postoperative falls, 2-year prosthetic complications (dislocation, mechanical loosening, periprosthetic fracture, and fibrosis), length of hospital stay, 90-day readmission rates, and time from index procedure to revision total knee arthroplasty.

Continuous variables are described using means ± standard deviation, and categorical variables are described using frequencies and percentages, where appropriate. Student’s *t*-test was utilized to assess differences in patient age, and Welch’s *t*-tests were used to assess differences in lengths of stay and time to revision. Chi-squared tests were used to assess differences in patient gender and comorbidities. A logistic regression model was created to calculate odds ratios (ORs) and 95% confidence intervals (CIs) on prior LLD diagnosis on fall rates, readmission rates, and implant-related complications. All statistical analyses were performed with R, version 4.2.1 software (R Foundation for Statistical Computation, Vienna, Austria). Statistical significance was set at an alpha of less than 0.05.

## Results

**Fig. 2:**
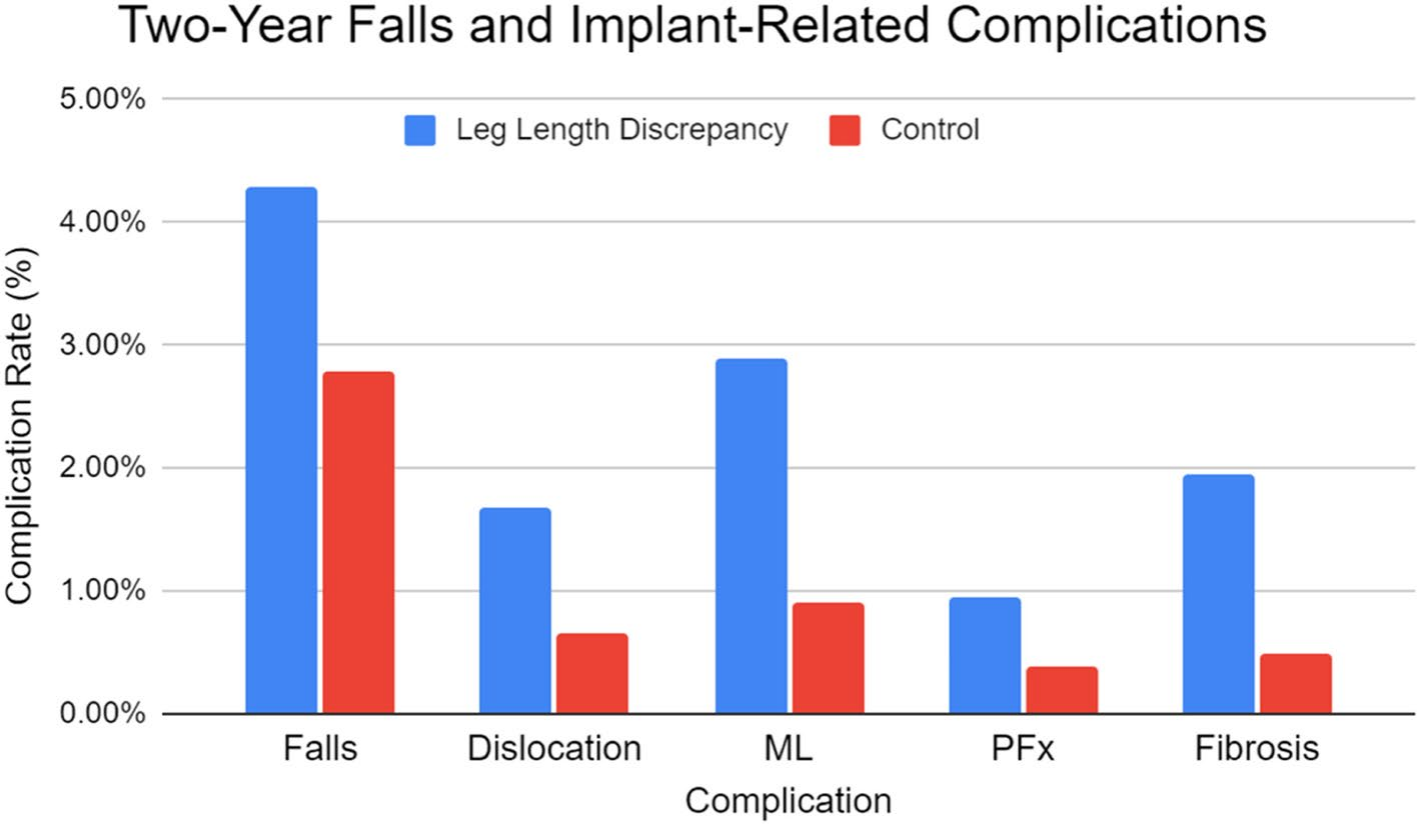
Two-year fall implant-related complication rate following total knee arthroplasty between patients with leg length discrepancy and matched-control patients

Rates of falls within 2-years postoperatively were significantly greater in the LLD group than in the control group (OR 1.56; 95% CI, 1.16–2.10; *P* = 0.003). Odds of all 2-year implant-related complications assessed were greater in the LLD group. The greatest increase in odds was with fibrosis (OR 4.03; 95% CI, 2.42–6.70; *P* < 0.001), followed by mechanical loosening (OR 3.29; 95% CI, 2.20–4.92; *P* < 0.001), and dislocation (OR 2.58; 95% CI, 1.56–4.28; *P* < 0.001) (Table 2 and Fig. 2).

**Table 2:** Two-year falls and implant-related complications between TKA patients with LLD and a matched-control cohort

Complication	LLD (%)	Control (%)	OR	95% CI	*P-*Value
Falls	4.28	2.79	1.56	1.16–2.10	**0.003**
DL	1.67	0.65	2.58	1.56–4.28	** < 0.001**
ML	2.90	0.90	3.29	2.20–4.92	** < 0.001**
PFx	0.94	0.38	2.51	1.29–4.90	**0.007**
Fibrosis	1.96	0.49	4.03	2.42–6.70	** < 0.001**

TKA, total knee arthroplasty; OR, odds ratio; CI, confidence interval; DL, dislocation; ML, mechanical loosening; PFx, periprosthetic fracture. Bold font signifies statistical significance

Hospital readmissions within 90 days postoperatively were not significantly different between study cohorts (6.97% vs. 6.01%, *P* = 0.178). In-hospital length of stay was significantly greater in the LLD group when compared to the control group (4.9 days vs. 3.0 days,* P* < 0.001) (Table 3). The incidence of revision knee arthroplasty was 4.86% in the LLD group, and 3.09% in the matched-control group. The mean time from index procedure to revision surgery was significantly decreased in the LLD group (392 ± 386 days) when compared to the control group (928 ± 964 days) (*P* < 0.001) (Table 4).

**Table 3:** Medical and healthcare utilization between TKA patients with LLD and a matched-control cohort

Utilization	LLD	Control	OR	95% CI	*P-*Value
Readmission	6.97%	6.01%	1.17	0.93–1.47	0.178
Length of Stay (d)^a^	4.9 ± 6.0	3.0 ± 1.4			** < 0.001**

^a^Given as mean ± standard deviation. Bold font signifies statistical significance

**Table 4:** Time to revision surgery between TKA patients with leg length discrepancy and a matched-control cohort

Utilization	LLD	Control	*P-*Value
Time to Revision (d)^a^	392 ± 386	928 ± 964	** < 0.001**

^a^Given as mean ± standard deviation. Bold font signifies statistical significance

## Discussion

In this retrospective matched-control study, patients who had LLD and underwent TKA had significantly greater rates of falls within 2 years, odds of prosthetic complications, longer length of hospital stay, and earlier time to revision than TKA patients with no prior diagnosis of LLD. The increase in fall rates that we observed is likely due to several factors in this patient population. Firstly, patients who have long-standing LLD are likely to develop compensatory strategies to accommodate their imbalance [15–17]. However, following TKA, these adaptive mechanisms may be disrupted which can affect the stability and coordination of movements, increasing the risk of stumbling or losing balance, particularly during the early stages of postoperative recovery. Secondly, LLD can result in muscle weakness and asymmetry. The muscles around the hip, thigh, and lower leg may be affected by the discrepancy, leading to differences in strength and function [14, 18]. This muscular imbalance can further contribute to instability and difficulties in controlling movements, making it harder for patients to maintain their balance and prevent falls. Lastly, living with a leg length discrepancy can lead to altered proprioceptive feedback and adaptation in the leg and hip joints [19]. Following TKA, the proprioceptive system needs time to readjust and recalibrate to the new limb length and alignment. This adjustment period can result in temporary deficits in proprioception, leading to instability and an increased fall risk [20].

Patients who have leg length discrepancy prior to their TKA were at significantly greater odds of all prosthetic complications assessed within 2 years postoperatively. Achieving proper implant alignment and stability in this population may be challenging. If limb length is not properly accounted for during preoperative planning or addressed during surgery, it can result in residual LLD, which can lead to implant instability, altered joint mechanics, and increased stress on the knee joint [21]. All of these factors can contribute to implant-related complications, such as instability, dislocation, premature polyethylene wear, and component loosening [22]. Long-term LLD can result in uneven tension and strain on the soft tissues, ligaments, and tendons surrounding the knee joint. During TKA, these imbalances may persist or become more pronounced if the soft tissue envelope is not adequately addressed. Persistent soft tissue imbalances can affect joint stability, compromise the function of the prosthesis, and increase the risk of implant-related complications [23, 24]. Finally, these patients may experience changes in walking patterns, joint load, and forces exerted on the knee joint. These altered biomechanics can put additional stress on the components, potentially leading to component wear, instability, or failure over time [21].

The increased length of stay in the LLD cohort may be due to the increased complexity of the surgical procedure, as additional steps, including bone resection or soft tissue release, may be required to address the LLD and achieve proper leg length alignment. These additional steps can prolong surgical time and increase blood loss, both of which have been shown to increase the length of stay [25]. Additionally, patients who have LLD may require more intensive and specialized rehabilitation after TKA to address the challenges associated with the discrepancy. Physical therapy and rehabilitation programs may need to focus on correcting gait abnormalities, restoring balance, and retraining muscles to accommodate the new leg length [26, 27].

Finally, the time from index procedure to revision surgery was significantly shorter in patients who had LLD prior to their TKA. This is likely due to the combination of increased fall risk and increased odds of implant complications discussed previously. This is an extremely important finding, as revision surgery is associated with elevated patient morbidity, increased costs, and places a significant burden on healthcare resources and utilization [28–31]. Revision knee arthroplasty carries higher risks of infection, thromboembolic complications, nerve or blood vessel injury, and even mortality [28]. Increased costs are multifactorial, including additional diagnostic testing, more intricate surgical technique, and specialized implants, as well as extended hospital stays [30]. Finally, revision surgery involves additional operating room time, specialized surgical teams, and more extensive postoperative care, including increased monitoring, medication, and rehabilitation services [31].

This study is however not without limitations. Many of these are inherent with the use of a large national database, and the retrospective nature of the study. First, statements of causality cannot be made when utilizing retrospectively gathered information. Secondly, PearlDiver is reliant on the proper input of codes including diagnoses and procedures. If there are errors in coding, then this potentially could introduce collection bias into the study. Third, we are unable to ascertain the degree or etiology of leg length discrepancy among patients, the duration from the time of their diagnosis to the time of their operation, or whether the limb with the length discrepancy was the operative leg or the contralateral limb. Additionally, whether LLD was corrected at the time of surgery was not assessed, which does not allow us to evaluate whether these results are secondary to persistent LLD. However, a strength of this study is that through the use of PearlDiver, over 157 million patient files were queried, allowing this study to have significant statistical power. To our knowledge, this is the first study to assess the effects that preoperative leg length discrepancy has on outcomes and complications following total knee arthroplasty.

## Conclusion

Leg length discrepancy in patients undergoing total knee arthroplasty is associated with significantly greater rates of falls, odds of prosthetic complications, longer length of hospital stay, and earlier time to revision. It may be prudent to evaluate limb length preoperatively so that surgical planning and perioperative care can be optimized for the patient to reduce the involved risks. The findings of this study justify future prospective and higher-level research to better understand the effects of limb length discrepancy prior to total knee arthroplasty.

### Supplementary Information

**Additional file 1: Supplemental Table 1.** International classification of disease (ICD), the ninth and tenth revision codes, and current procedural terminology (CPT) codes utilized to identify patients, procedures performed, diagnoses, and complications.

## Data Availability

The datasets used and/or analyzed during the current study are available from the corresponding author upon reasonable request. Original data in the form of articles used in this review are available with public access.

## Abbreviations

LLD: Leg length discrepancy
TKA: Total knee arthroplasty
HIPAA: Health Insurance Portability and Affordability Act
ICD: International Classification of Disease
CPT: Current Procedural Terminology
OR: Odds ratio
CI: Confidence interval
