# Supplementary material for: Leg length discrepancy before total knee arthroplasty is associated with increased complications and earlier time to revision
Source: Arthroplasty. 2024 Jan 16;6:5. doi: 10.1186/s42836-023-00221-3 (PMC10790485; doi:10.1186/s42836-023-00221-3)
Supplement: Supplementary file 1 — Additional file 1: Supplemental Table 1. International classification of disease (ICD), the ninth and tenth revision codes, and current procedural terminology (CPT) codes utilized to identify patients, procedures performed, diagnoses, and complications. [file 42836_2023_221_MOESM1_ESM.docx]

**Supplemental Data**

**Supplemental Table 1.** International classification of disease (ICD), the ninth and tenth revision codes, and current procedural terminology (CPT) codes utilized to identify patients, procedures performed, diagnoses, and complications.

| Description | Codes Used |
| --- | --- |
| Total Knee Arthroplasty | CPT-27447, CPT-27445, ICD-9-P-8154, ICD-10-P-0SRT0J9, ICD-10-P-0SRT0JA, ICD-10-P-0SRT0JZ, ICD-10-P-0SRU0J9, ICD-10-P-0SRU0JA, ICD-10-P-0SRU0JZ, ICD-10-P-0SRV0J9, ICD-10-P-0SRV0JA, ICD-10-P-0SRV0JZ, ICD-10-P-0SRW0J9, ICD-10-P-0SRW0JA, ICD-10-P-0SRW0JZ |
| Leg Length Discrepancy | ICD-10-D-M21751, ICD-10-D-M21752, ICD-10-D-M21761, ICD-10-D-M21762, ICD-10-D-M21763, ICD-10-D-M21764 |
| Falls | ICD-9-D-E8881, ICD-9-D-E8889, ICD-10-D-W19XXXA, ICD-10-D-W19XXXD |
| Implant-Related Complications | Prosthetic joint infection: ICD-9-D-99666, ICD-9-D-99669, ICD-10-D-T8450XA, ICD-10-D-T8450XD, ICD-10-D-T8450XS, ICD-10-D-T8579XA, ICD-10-D-T8579XD, ICD-10-D-T8579XS  Mechanical loosening: ICD-9-D-99641, ICD-10-D-T84032, ICD-10-D-T84039S  Periprosthetic fracture: ICD-9-D-99644, ICD-10-D-M971, ICD-10-D-M9712XS, ICD-10-D-M979, ICD-10-D-M979XXS Dislocation: ICD-9-D-99642, ICD-10-D-T84022, ICD-10-D-T84029S  Fibrosis: ICD-10-D-T8482, ICD-10-D-T8482XS |
| Revision Knee Arthroplasty | CPT-27486, CPT-27487, ICD-10-P-0SWC08, ICD-10-P-0SWC0KZ, ICD-10-P-0SWD08, ICD-10-P-0SWD0K, ICD-10-P-0SWT0JZ, ICD-10-P-0SWU0JZ, ICD-10-P-0SWV0JZ, ICD-10-P-0SWW0JZ |
